# A Method for Predictive Analysis of Platelet Supply

**DOI:** 10.1007/s12288-025-02083-y

**Published:** 2025-07-10

**Authors:** Changhong Kong, Junna Qiu, Yebiao Xu, Cuie Wang, Kaili Wu, Risheng He, Wei Hu

**Affiliations:** Blood Center of Zhejiang Province, 789 Jianye Road, Binjiang District, Hangzhou, 310052 China

**Keywords:** Platelet supply, Time series analysis, Decomposition-combination forecast model, Rolling window, Weighted MAPE

## Abstract

**Background:**

In the realm of medical management, effectively resolving the disparity between blood supply and demand is fundamental to the management of the platelet supply chain. Central to this is the accurate estimation and prediction of blood supply that is instrumental in conserving resources and minimizing expenses. However, time series analyses that rely on single models struggle to capture the intricacies of changing demand structures of blood and are susceptible to external events, which can lead to distorted forecasts. Moreover, the common pitfalls of conventional model evaluation methods, such as their short-term focus and one-sidedness, hinder the accurate assessment of a model's performance in the field of prediction of blood supply.

**Objective:**

This paper aims to construct a more comprehensive platelet clinical supply forecasting system by integrating the strengths of various prediction models and enhancing evaluation methodologies.

**Methods:**

This paper introduces a novel Decomposition-Combination Prediction Model that leverages X-13ARIMA-SEATS, where the individual components are modeled using ARIMA, TimeGPT, and SNAIVE methodologies. To assess the model's performance, evaluation metrics are meticulously constructed using a rolling window approach coupled with exponential decay weighting, allowing for a more nuanced evaluation through weighted MAPE. The efficacy and robustness of this approach are subsequently validated using platelet data from the Zhejiang Blood Center, providing a rigorous test of the method's practical applicability.

**Results:**

Empirical analysis demonstrates that the decomposition-combination model outperforms the individual ARIMA, Prophet, and TimeGPT models in terms of forecasting accuracy. Furthermore, sensitivity analysis reveals that while the decay factor influences the weighted MAPE results, the overall assessment of model performance remains robust.

**Conclusions:**

The decomposition-combination model adeptly captures the intrinsic characteristics of platelet series, thereby enhancing the model's forecasting accuracy. Concurrently, the rolling window-based weighted MAPE evaluation method effectively discerns the influence of external events and accurately assesses the forecast performance, thereby enhancing the model's generalization capabilities.

## Introduction

Platelet transfusions are a crucial component of contemporary medical practice, employed both prophylactically to mitigate the risk of bleeding and therapeutically to address active hemorrhages [1]. In recent years, there has been a significant surge in the clinical demand for platelets in impacted regions. Consequently, many blood centers are striving to maintain substantial inventories of platelets and other blood components [1–4]. However, the limited shelf life of platelets poses a dual challenge: while excessive inventory can lead to waste from expiration, insufficient stock increases the risk of supply shortages. This dilemma presents significant difficulties for blood centers in managing platelet collection and distribution [5]. Thus, achieving an accurate forecast of platelet demand is fundamental and pivotal to optimizing the management of the platelet supply chain.

In the realm of platelet forecasting, current research often relies on univariate time series models such as weighted moving averages and Holt-Winters exponential smoothing methods [6–8]. These models encounter challenges in identifying and decomposing the underlying factors that influence time series trends, or they may lack the granularity needed to accurately describe the characteristics of these decomposed components, resulting in potential prediction biases. Furthermore, while the traditional ARIMA model [6], based on the Box-Jenkins methodology, can achieve more accurate forecasts, it is not without its drawbacks. These include the inability to automatically select globally optimal parameters and a vulnerability to unforeseen external events. Additionally, the model's typically limited forecasting scope means that existing evaluation methods, particularly those based on MAPE [9], have inherent limitations in assessing a model's comprehensiveness and generalization capabilities, not to mention their inability to fully discern the impact of unconventional factors. Consequently, this paper aims to construct a more comprehensive platelet clinical supply forecasting system by integrating the strengths of various prediction models and enhancing evaluation methodologies.

This paper employs a decomposition-combination approach utilizing the X-13ARIMA-SEATS [10] method to dissect a single time series into three core components: trend, seasonal, and noise. A variety of forecasting techniques, including ARIMA, TimeGPT [11], SNAIVE [12], and the simple MEAN, are then applied to fit these individual series. Concurrently, the Hyndman-Khandakar algorithm is deployed to automate the parameter search process, elucidating the underlying factors of the complex time series. Furthermore, to provide a more holistic assessment of the model's forecast performance across various time frames and to discern the effects of exceptional time points, a rolling window sample is selected to calculate the model's forecast error. This error is weighted by an exponential decay function, yielding a weighted average error indicator based on MAPE. Ultimately, platelet data from the Zhejiang Blood Center is utilized to substantiate the efficacy and practicality of the proposed model.

## Methods

Currently, the majority of mainstream studies in blood supply forecasting rely on single-fitting models like exponential smoothing and ARIMA to predict time series data, which are capable of capturing the general trends of these series. However, these models struggle to accurately describe sequences that undergo significant short-term changes due to the impact of unconventional factors, such as specific years, external events, and contingencies. To address this limitation, this paper introduces the X-13ARIMA-SEATS model to decompose the original time series. Subsequently, ARIMA, TimeGPT, SNAIVE, and MEAN models are employed to fit the primary components of the series. This method aims to gain a deeper understanding of how the three components derived from series decomposition influence the overall trend across different time intervals.

**Fig. 1:**
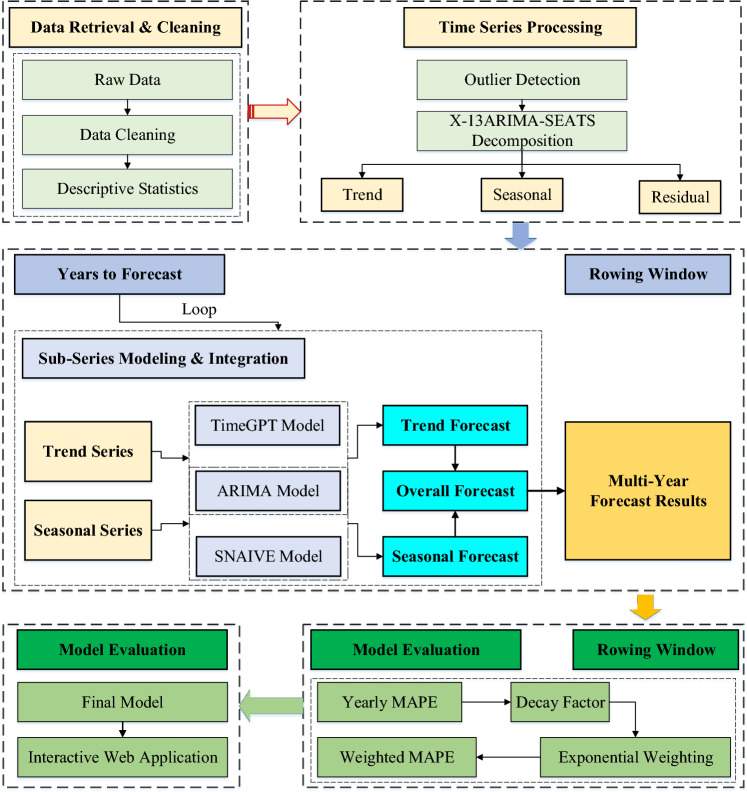
Research process and design idea

Furthermore, traditional model performance evaluation methods fall short in assessing the comprehensiveness and generalization capabilities of forecast models, and they are ineffective at pinpointing the impact of unconventional factors. To address these shortcomings, this paper adopts the rolling window approach, constructing evaluation metrics from continuous samples obtained through this method. By employing an exponential decay weighting technique to calculate the weighted MAPE, we aim to thoroughly examine the entire modeling process, thereby enhancing the objectivity and precision of our evaluation outcomes. The research process and design philosophy are depicted in Fig. 1.

### Decomposition-Combination Forecast Model Based on X-13ARIMA-SEATS

The X-13ARIMA-SEATS model is a sophisticated statistical framework that builds upon the foundations of X-11 and ARIMA, while incorporating the strengths of the SEATS approach. It is particularly adept at handling economic and statistical data that exhibit seasonal, trend, and cyclical patterns [10]. This model posits that a time series (*Y*_*t*_) can be meticulously decomposed into three distinct components: the trend term (*T*_*t*_), the seasonal term (*S*_*t*_), and the residual term (*I*_*t*_), which are specifically expressed as $Y_{t} = T_{t} \times S_{t} \times I_{t}$.

Further, a variety of methods are employed to fit each component derived from the decomposition process. Specifically, the trend series can be fitted using ARIMA and TimeGPT approaches, the seasonal series can be addressed with ARIMA and SNAIVE techniques, and the residual series can be captured by ARIMA and MEAN methods. Finally, the predicted values $\hat{T}_{t}$, $\hat{S}_{t}$ and $\hat{I}_{t}$ of the decomposed series are obtained respectively. Additionally, the Hyndman-Khandakar algorithm is employed to automatically identify the optimal parameters for the ARIMA model that need to be estimated. Subsequently, the forecasting of the time series is $\hat{Y}_{t} = \hat{T}_{t} \times \hat{S}_{t}$.

### Evaluation Method Based on Rolling Window

In conventional blood supply forecasting research, the scope of future trend predictions is typically confined to the short term, with model effectiveness assessed over a brief prediction period. However, this approach has its limitations, as it relies solely on a single set of training data to build the model, resulting in a limited evaluation horizon. Moreover, the forecast model's robustness is vulnerable to fluctuations during exceptional periods, and its generalization capabilities are relatively weak. To enhance the model's representativeness and render the evaluation outcomes more objective and precise, we introduce the rolling window method. This method allows for the construction of corresponding prediction models through continuously selected samples via the rolling window technique, and the calculation of weighted Mean Absolute Percentage Error (MAPE). This not only aids in identifying specific temporal anomalies but also facilitates a more rigorous evaluation and selection of models.

**Fig. 2:**
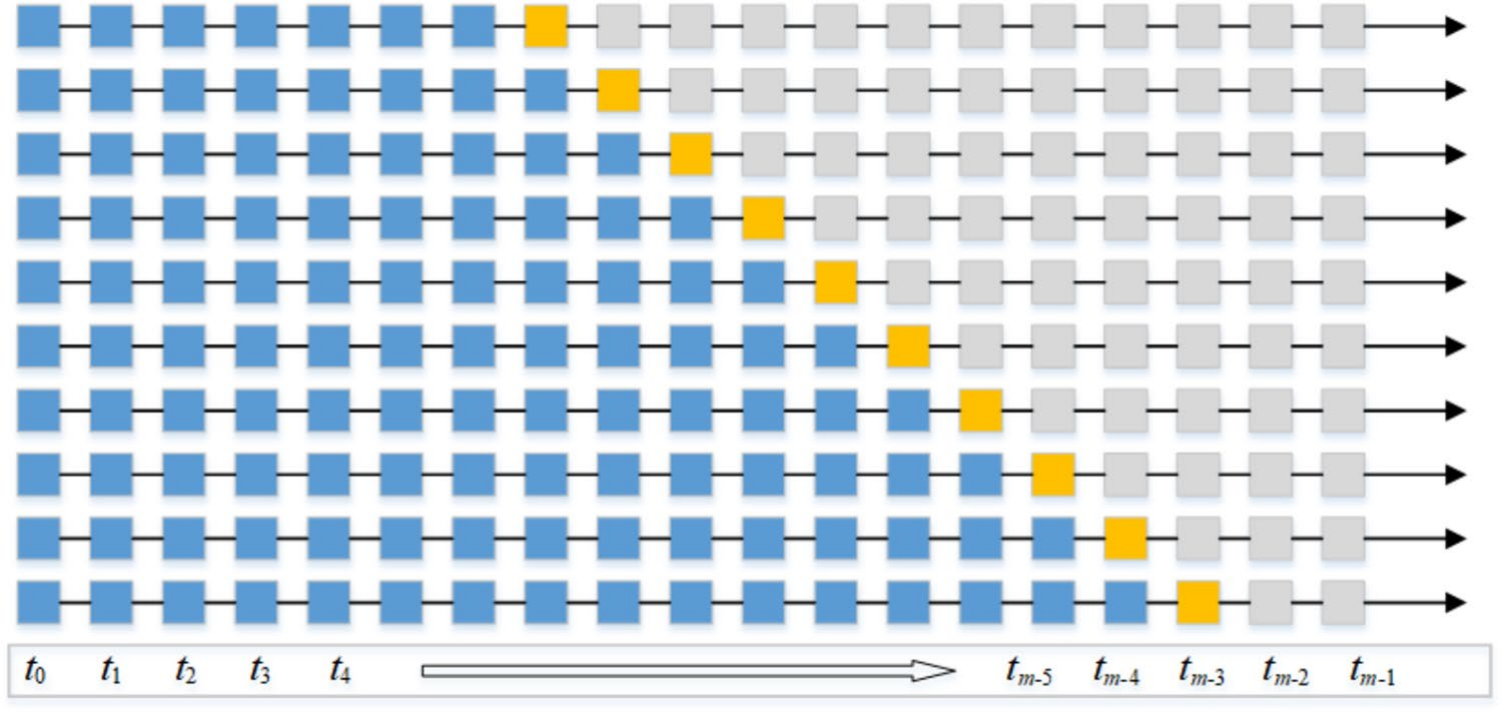
Time series analysis based on rolling window diagram

Rolling window analysis involves incrementally shifting the data window according to a predefined step size and subsequently analyzing the data captured within each window, as illustrated in Fig. 2. The blue dots denote the training dataset, while the orange dots signify the prediction dataset. Furthermore, when deriving the comprehensive evaluation index through rolling window analysis, the forecasting errors at each time point are adjusted by an exponential decay weight. This process allows for the calculation of the weighted Mean Absolute Percentage Error (MAPE), which is formulated as follows:

$$WeightedMAPE = \sum\limits_{{t = t_{0} }}^{{t_{m} }} {\left( {\frac{{\alpha^{{t_{m} - t}} }}{{\sum\nolimits_{{t = t_{0} }}^{{t_{m} }} {\alpha^{{t_{m} - t}} } }}MAPE_{t} } \right)}$$.

*t* is the predicted year, *t*_0_ is the initial year, *t*_*m*_ is the final year, $\alpha$ is a decay factor. This weighting approach places greater emphasis on the more recent years while also considering the annual forecast performance of the model.

## Results

**Fig. 3:**
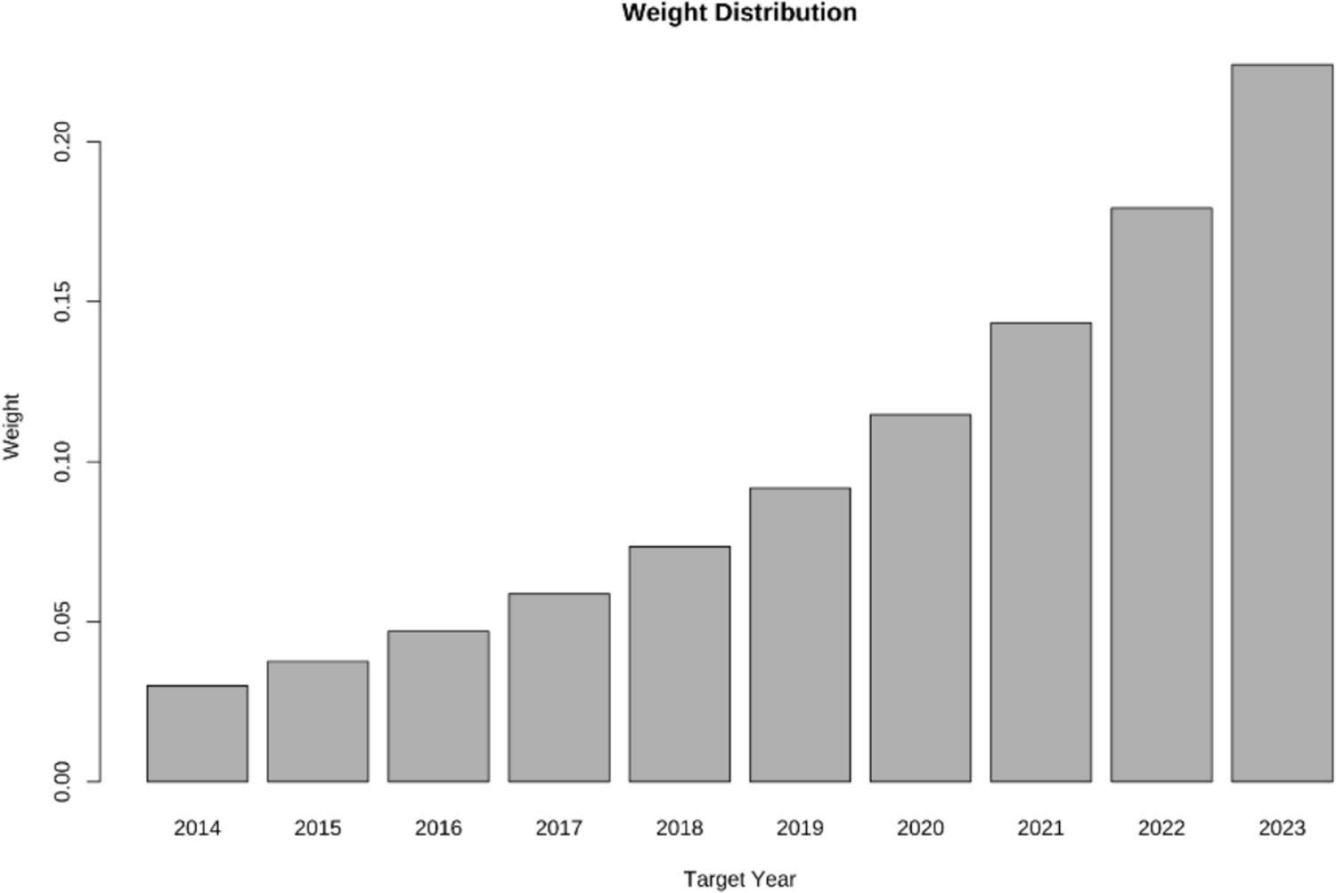
Weight distribution diagram of weighted MAPE

The empirical analysis is grounded in monthly platelet supply data from the Zhejiang Blood Center, spanning from January 2006 to December 2023. Considering the data's structural characteristics, we have determined a rolling window step length of 12 months and a window width of 60 months. Regarding the exponential decay weighting method, a smaller decay factor results in a broader weight range and an uneven distribution of weights. Furthermore, taking into account the sensitivity analysis outcomes presented in section "Sensitivity And Comparative Analysis", we have chosen a decay factor as $\alpha { = }0.8$. Consequently, the resulting weight distribution is illustrated in Fig. 3.

**Fig. 4:**
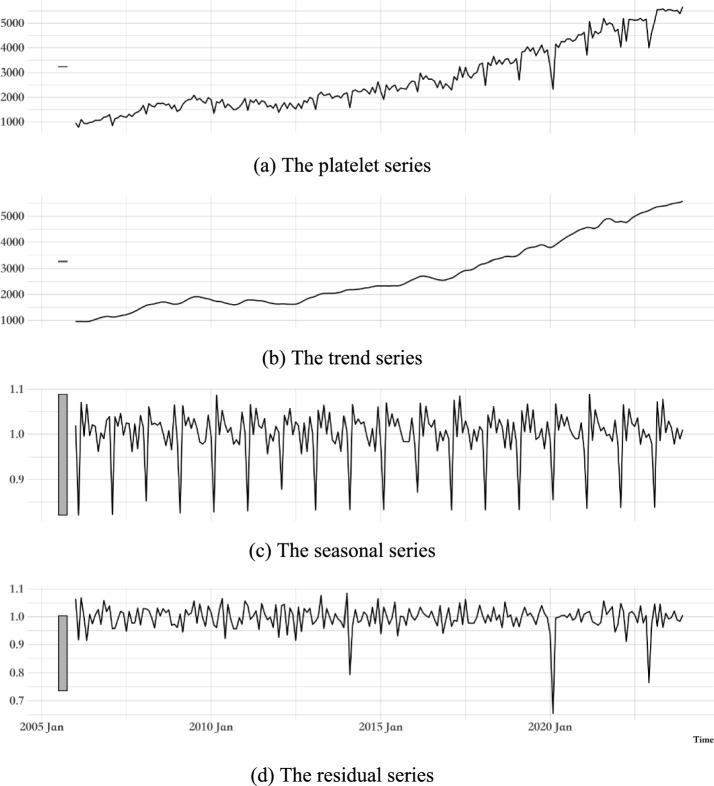
Platelet series and its decomposed components

We employ the X-13ARIMA-SEATS method from R software's seasonal package to analyze the platelet supply data. An exponential transformation is applied to stabilize the variance within the series. The components of trend (b), seasonal (c), and residual (d) are extracted, with the Chinese New Year serving as an exogenous variable in the analysis. The resulting decomposed components are depicted in Fig. 4.

Figure 4 illustrates that the trend series (b) exhibits a generally steady upward trajectory, and the seasonal series (c) demonstrates a relatively stable pattern overall. Concurrently, the residual series (d) resembles white noise, yet it experienced a pronounced fluctuation in the early stages of the Covid-19 pandemic and significant variations throughout the duration of the outbreak.

Regarding model selection, ARIMA and TimeGPT are deployed to fit the trend series (b), while ARIMA and SNAIVE are utilized to capture the seasonal patterns within the series (c). Concurrently, ARIMA in conjunction with MEAN is employed to analyze the residual series (d). The weighted MAPE results, which provide a quantitative assessment of these models, are presented in Table 1.

**Table 1:** Weighted MAPE of decomposed series based on different models

Components	ARIMA(A)	TimeGPT(T)	SNAIVE(S)	MEAN(M)
Trend Series (b)	**2.562%**	3.050%		
Seasonal Series (c)	**1.084%**		1.120%	
Residual Series (d)	3.305%			**3.279%**

A, T, S and M denote ARIMA, TimeGPT, SNAIVE and MEAN, respectively

Bold represents the result of the minimum weighted MAPE results

Examining the predictive outcomes, it is evident that the ARIMA model excels in forecasting trends and seasonal series, whereas the MEAN model demonstrates superior performance with residual series. Furthermore, the weighted MAPE for the platelet series (a), is derived from the synergistic integration of series forecasts from various models, as detailed in Table 2.

**Table 2:** Weighted MAPE based on decomposition-composition model under rolling window

Year	A(*T*) × S(*S*) (%)	A(*T*) × A(*S*) (%)	T(*T*) × S(*S*) (%)	T(*T*) × A(*S*) (%)
2014	4.360	4.451	4.189	4.129
2015	3.745	3.890	4.478	4.503
2016	6.557	6.239	5.033	4.662
2017	3.787	3.934	11.002	11.316
2018	5.048	5.122	3.410	3.805
2019	6.188	6.351	6.162	6.326
2020	**10.548**	**10.651**	**9.036**	**9.142**
2021	4.782	4.868	5.144	5.062
2022	5.413	5.383	5.335	5.259
2023	3.609	3.366	3.581	3.323
Weighted MAPE	**5.416**	**5.402**	**5.526**	**5.499**

A(T) × S(S) denotes a combined model whose trend series predicted by ARIMA and seasonal series predicted by SNAIVE, and the rest of the same

Bold represents the result of the minimum weighted MAPE results

As observed in Table 2, the weighted MAPE for the total series forecasting outcomes, derived from the integration of trend and seasonal components across various models, consistently falls within the range of 5.4% to 5.6%. Among these, the ARIMA (Trend) × ARIMA (Seasonal) model demonstrates the most superior forecasting accuracy, with the lowest weighted MAPE at 5.402%. Furthermore, when examining the weighted MAPE for forecasting results across previous years, the year 2020 emerges as an exceptional time point. The weighted MAPE for the forecasting results in 2020 is notably higher, ranging from 9.0% to 11.0%, suggesting that the pandemic has a significant impact on the prediction of platelet supply dynamics.

## Sensitivity and Comparative Analysis

In the empirical analysis, we posit a decay factor $\alpha$ as 0.8 to underpin our study. To mitigate the risk of outcome contingency, a sensitivity analysis regarding the decay factor is imperative. We explore a spectrum of decay factor values, ranging from 0.1 to 0.9 in increments of 0.1. Consequently, we compute the corresponding weighted MAPE for each of the four combined models, as detailed in Table 3.

**Table 3:** The variation of weighted MAPE with decay factor

Decay Factor	A(*T*) × S(*S*) (%)	A(*T*) × A(*S*) (%)	T(*T*) × S(*S*) (%)	T(*T*) × A(*S*) (%)
0.1	3.788	3.568	3.758	**3.518**
0.2	3.983	3.788	3.950	**3.730**
0.3	4.211	4.043	4.169	**3.971**
0.4	4.473	4.335	4.420	**4.249**
0.5	4.758	4.653	4.701	**4.561**
0.6	5.038	4.965	4.999	**4.896**
0.7	5.270	5.229	5.287	**5.224**
0.8	5.416	**5.402**	5.526	5.499
0.9	**5.456**	5.463	5.680	5.681

Bold represents the result of the minimum weighted MAPE results

Table 3 reveals that the decay factor exerts a significant influence on the performance assessment of forecasting models to a certain degree. Across various models, an increase in the decay factor leads to a corresponding reduction in the variance of annual weights, promoting a more balanced distribution of weights and a consequent gradual increase in the weighted MAPE. When the decay factor ranges from 0.1 to 0.7, the T(*T*) × A(*S*) combination model demonstrates the most favorable performance, with the A(*T*) × A(*S*) combination model following closely behind, exhibiting a weighted MAPE that is only approximately 0.1% higher than the former. At a decay factor of 0.8, the A(*T*) × A(*S*) combination model emerges as the most effective. However, at a decay factor of 0.9, the A(*T*) × S(*S*) combination model achieves the best predictive performance, with the A(*T*) × A(*S*) combination model ranking second. Thus, it is evident that the A(*T*) × A(*S*) combination model maintains a relatively stable and robust forecast performance across different decay factors.

Furthermore, to showcase the strengths of the decomposition-combination forecasting approach, the ARIMA, Prophet [13], and TimeGPT models have been deployed to individually model and forecast platelet supply. Subsequently, the MAPE and weighted MAPE outcomes for each year, derived from these models, are compared with those of the X-13ARIMA-SEATS model. The comparative results are presented in Table 4.

**Table 4:** Weighted MAPE based on different models under rolling window

Year	X-13ARIMA-SEATS (%)	ARIMA (%)	Prophet (%)	TimeGPT (%)
2014	**4.451**	20.547	5.033	6.876
2015	**3.890**	6.057	6.676	4.857
2016	6.239	7.704	**5.829**	7.886
2017	**3.934**	13.543	6.147	11.112
2018	**5.122**	6.550	6.412	10.066
2019	6.351	6.567	**4.027**	10.924
2020	10.651	8.018	**8.933**	10.402
2021	**4.868**	7.422	5.041	9.678
2022	**5.383**	5.948	7.175	5.677
2023	**3.366**	6.775	5.039	14.901
Weighted MAPE	**5.402**	7.655	6.040	10.092

Bold represents the result of the minimum weighted MAPE results

Table 4 reveals that when evaluated using weighted MAPE as the benchmark, the TimeGPT model exhibits the poorest forecasting accuracy, with the general ARIMA model following closely behind. The Prophet model outperforms these two models, attributable to its capability for series decomposition. However, the X-13ARIMA-SEATS model surpasses all three in terms of forecasting accuracy, owing to its component-based modeling approach. Furthermore, the X-13ARIMA-SEATS model demonstrates a relatively strong performance in annual forecasting, with only minor deficits compared to the Prophet model in the years 2016, 2019, and 2020. As mentioned, leveraging the information from time decomposed components for fitting and prediction is instrumental in enhancing the model's forecasting capabilities.

## Discussion

As socio-economic development and urbanization keep moving forward, the need for blood in hospitals is going up. However, the number of people donating blood hasn't been able to catch up with this growing need, resulting in a larger gap between how blood demand and supply. This imbalance causes much pressure on blood centers. Moreover, the impact of external events such as the COVID-19 pandemic have made it even harder to keep the blood supply stable. Traditional methodologies of analyzing time series often have disadvantages on describing the changing trend of blood demand, and existing evaluation metrics for assessing the performance of model lacks comprehensiveness. These deficiencies lead to unavailable assessment of model’s forecasting effectiveness. Under this backdrop, this paper undertakes a predictive analysis of platelet supply at the Zhejiang Blood Center. It employs a novel forecasting model and a robust evaluation methodology to address these challenges.

In this paper, we come up with a new forecasting methodology based on a decomposition-combination model with a rolling window mechanism. First, trend, seasonality, and residuals parts are dissected from the platelet supply series using X-13ARIMA-SEATS model, which respectively reflect the long-term trend, periodic variation and random variation of platelet data. The trend and seasonal components are then modeled using ARIMA, TimeGPT, SNAIVE, and MEAN models, each selected for thier suitability in capturing the characteristics of trend and seasonal patterns extracted from platelet data. Simultaneously, we progressively adjust the size of the rolling window, which enables the dynamic analysis and evaluation of the platelet data as window advances. Each year's data is assigned an exponentially decreasing weight, and the model forecasting efficacy is measured through a weighted MAPE. By applying proposed method, we conduct some experiments and comparisons to demonstrate our decomposition-combination model is more accurate than other single models (ARIMA, Prophet, TimeGPT), particularly when fitting the platelet data from 2021 to 2023 during the COVID-19 pandemic, it illustrates a more remarkable accuracy. In addition, a sensitivity analysis also shows that while the decay factor can slightly influence the weighted MAPE results, while the performance assessment of proposed model remains robust and reliable.

The time series decomposition-combination forecasting model introduced in the aforementioned, enhanced with the rolling window evaluation approach, has significantly extended the ability to predict blood data beyond just platelet counts. It has potential for forecasting different types of blood, such as total blood volume, red blood cells, and plasma supplies. In the future, research may explore various time series decomposition methods to capture patterns beyond the conventional trend-season-residual paradigm. In addition, the decomposed components could be analyzed with advanced machine learning techniques like LSTM, XGBoost, and LightGBM. Simultaneously, incorporating auxiliary variables may further improve VAR models, while GARCH models could be adapted to handle the decomposed residual series, making it easier to deal with heteroscedasticity issues in the blood supply data.

## Data Availability

The data sets generated during or analyzed during this study are available from the corresponding author on reasonable request.
